# Peptides whose levels change during the attack period in episodic migraine: NETRIN-1 and amylin

**DOI:** 10.3389/fneur.2026.1932356

**Published:** 2026-09-03

**Authors:** Aysenur Ince Kocoğlu, Mustafa Altas, Turan Akdag, Ahmet Bugrul, Mehmet Uyar

**Affiliations:** 1 Neurology Department, Necmettin Erbakan University Hospital, Konya, Türkiye; 2 Neurology Department, Kelkit State Hospital, Gumushane, Türkiye; 3 Medical and Cosmetic Plant Application and Research Center, Necmettin Erbakan University Meram Vocational School, Konya, Türkiye; 4 Public Health Department, Necmettin Erbakan University Hospital, Konya, Türkiye

**Keywords:** amylin, episodic migraine, migraine with aura, Netrin-1, Neuroinflammation

## Abstract

**Objectives:**

The role of neuroinflammation in the pathophysiology of migraine is becoming increasingly important. The aim of this study was to evaluate serum netrin-1 and amylin levels in patients with episodic migraine.

**Methods:**

The study included 60 patients diagnosed with episodic migraine and 30 healthy controls. Patients were evaluated during an ictal (*n* = 25) or interictal (*n* = 35) period. They were further categorized based on the presence of aura (migraine with aura, *n* = 25; migraine without aura, *n* = 35).

**Results:**

Serum netrin-1 levels were markedly lower in the migraine group compared to the control group (492.7 ± 638.0 pg/mL vs. 744.8 ± 754.7 pg./mL, *p* = 0.038). Although amylin levels were also lower in migraine patients (324.9 ± 451.7 ng/mL vs. 453.7 ± 490.2 ng/mL), this difference did not reach statistical significance (*p* = 0.141). No significant differences in netrin-1 and amylin levels were observed between patients with and without aura, nor between ictal and interictal periods. In the ROC analysis, netrin-1 levels demonstrated significant diagnostic performance in distinguishing migraine patients from controls (AUC = 0.635; 95% CI: 0.512–0.758; *p* = 0.031). The ROC analysis for amylin was not significant (AUC = 0.596, *p* = 0.133), and the combined model yielded an AUC of 0.636 (*p* = 0.036).

**Conclusion:**

Serum netrin-1 levels were markedly lower in patients with episodic migraine compared with healthy controls, while amylin levels also showed a decreasing trend. Further large-scale studies are needed to elucidate the clinical significance and pathophysiological roles of these biomarkers in migraine.

## Introduction

Migraine is a common neurological disorder affecting one in four women and one in ten men, with a total prevalence of 15.1% of the global population. Considering the young population affected by migraine and this population’s contribution to the economy, migraine holds an important place among neurological disorders due to its negative impact on the workforce during periods of headache. In most cases, migraine attacks are infrequent; however, some individuals experience a progressive increase in attack frequency over time. This condition can lead to chronification and affects approximately 2% of the general population. According to the International Classification of Headache Disorders, 3rd edition (ICHD-3), episodic migraine is defined as migraine occurring on fewer than 15 headache days per month, whereas chronic migraine is characterized by headache occurring on 15 or more days per month for more than 3 months, with at least 8 monthly headache days fulfilling migraine criteria or responding to migraine-specific treatment. The underlying mechanisms of chronicity have not yet been resolved, but it is accepted that inflammation plays a role to some extent. This condition is thought to be caused by the activation of protein kinases and increased expression of cytokines in trigeminovascular system neurons and glial cells, which is likely due to the presence of neurogenic neuroinflammation (1). Although the pathophysiology of trigemino-vascular system activation and the mechanisms that trigger it have not yet been fully elucidated, the prevailing view among various hypotheses is that it is primarily the result of thalamocortical dysrhythmia (2, 3).

Netrin-1 is a member of the laminin-like protein family and was initially defined as a potent chemoattractant. Studies indicate that it plays a role in cell migration during embryonic development. It is thought to be effective in inflammation, tissue remodeling, angiogenesis and cancer regulation. The protective or pathogenic effect of netrin-1 suggests that it could be a potential therapeutic target in many diseases (4). Netrin-1, a canonically secreted guidance cue, has been defined as a dual-function neuronal guidance molecule that interacts with receptors to direct axonal extension and is a protein that supports synapse formation during early development. As a bimodal protein, netrin-1 can bind to different receptors and generate two distinct responses depending on protein levels (5). Results from a series of studies on endogenously secreted netrin-1 have concluded that netrin-1 inhibits the movements of monocytes, lymphocytes and neutrophils through the activation of its receptors, thereby affecting immune responses. Recent clinical research findings have revealed a significant downregulation of netrin-1 in various autoimmune and inflammatory diseases (6).

Amylin (islet amyloid polypeptide) is produced and secreted by beta cells in the pancreas along with insulin. Amylin and CGRP (Calcitonin gene-related peptide) are structurally related peptides belonging to the calcitonin peptide family, sharing some receptors and bioactivities. Amylin and amylin receptors are found in different formations involved in pain modulation in the context of migraine, including the trigeminal-vascular system. Moreover, a limited number of preclinical studies have shown that amylin may have vasodilator and pronociceptive properties (7). It has been shown that an infusion of pramlintidine, an analog of a certain agent, triggers migraine attacks in nearly half of migraine patients and causes headaches in 88 percent of these patients. When evaluated in terms of its activity on receptors, this suggests that pramlintide’s ability to generate migraine attacks is likely independent of both CGRP and the CGRP receptor (8).

In our study, the concept of inflammation was examined in episodic migraine patients based on the concept of neuroinflammation, and factors such as migraine frequency and severity that affect the variability of molecules involved in this process among patients were investigated. Netrin-1 and amylin levels in episodic migraine patients were evaluated in attack, non-attack, and control group.

## Methods

This study was designed as a single-center, case–control observational study, and cross-sectional analysis conducted to evaluate the association between migraine and biomarkers whose elevated at attack, netrin-1 and amylin. Patients experiencing episodic migraine who presented at neurology outpatient clinic of a tertiary university hospital between January–April 2024 and healhty volunteers were included in the study. Migraine patients were enrolled from a neurology outpatient clinic and diagnosed based on the International Classification of Headache Disorders (ICHD-3) criteria. The patient diagnosed by a neurologist that newly and previously, either with aura (MWA) or without aura (MWoA). Patients were evaluated based on age, gender, BMI, headache severity assessed using scales according to the clinical characteristics of the headache and the degree to which it affected daily life, presence of aura, and dietary and sleep habits. Patients’ functional status was assessed based on their MIDAS (Migraine Disability Assessment) and HIT-6 (Headache Impact Test) scores over the past 3 months. In addition, the severity of headaches during attack episodes was assessed using the VAS (Visual Analog Scale). The VAS score was scored using a visual pain scale, while the MIDAS and HIT-6 scores were translated into Turkish, explained to patients in the form of completed studies, and scored (9, 10). People who walked for at least 20 min 3 days a week were considered to be engaging in regular exercise (11).

The study was undertaken and it conforms to the provisions of the Declaration of Helsinki (as revised in 75th WMA General Assembly, Helsinki, Finland, October 2024). Ethical approval was obtained from the Necmettin Erbakan University non-drug and non-medical device research ethics committee (decision number 2023/4431). The study and the parameters to be analyzed in the serum were explained to the patients and volunteers, and informed consent forms were obtained from each participant.

Twenty-five patients were classified as having attacks, 35 were non-attack, and 30 were healthy controls. Serum levels of netrin-1 and amylin obtained from venous blood samples after centrifugation were evaluated and compared with the specified characteristics. Patients who presented within 24 h of the onset of headache were considered to be in the attack phase, and blood samples were collected after a minimum fasting period of 2 h. Patients experiencing headaches similar to their previous migraine headaches within 24–72 h of the onset of pain were classified as being in the ictal phase, while patients diagnosed with episodic migraine who were not experiencing headache symptoms and for whom at least 48 h had elapsed since the last attack were classified as being in the interictal phase (12). Volunteers who visited the outpatient clinic for reasons other than headache complaints, who had never experienced migraine headaches in their lives, who did not smoke, and who had not taken nonsteroidal anti-inflammatory drugs within the last week were determined to be a healthy control group consistent with the age of the patient group.

Our study excluded patients under the age of 18, patients and volunteers with medication overuse headaches, primary headaches other than migraine, secondary headaches, pregnant women, menstruating patients, patients using CGRP antagonists for migraine prophylaxis, vasculitis, rheumatological or autoinflammatory diseases, active infection phase, malignancy, patients who have used simple analgesics, triptans or nonsteroidal anti-inflammatory drugs for headaches in the last 3 days, patients using sedative/analgesic drugs for headaches, and patients using antidepressants.

Serum samples were obtained using standard procedures, collected in polypropylene tubes, centrifuged to separate the serum, and stored immediately at −80 °C. Netrin-1 and amylin levels in serum were measured using BT LAB Human Netrin-1 (Ntn1) and BT LAB Human Islet Amyloid Polypeptide (IAPP) Amylin enzyme-linked immunosorbent assay (ELISA) kits according to the manufacturers’ instructions. Since no cutoff values were established for Netrin-1 and amylin levels, numerical values were used.

### Statistical analysis

Statistical analyses were performed using Jamovi (Version 2.6.45.0). Descriptive statistics for continuous variables were expressed as mean ± standard deviation or median (interquartile range), as appropriate, while categorical variables were expressed as frequencies and percentages. The Mann–Whitney *U* test was used to compare continuous variables between two groups. Receiver operating characteristic (ROC) curve analysis was conducted to evaluate the diagnostic performance of Netrin-1 and amylin. Sensitivity, specificity, positive predictive value (PPV), and negative predictive value (NPV) were calculated based on 2*2 contingency tables at the optimal cut-off values. The Spearman correlation test was used to evaluate relationships between quantitative variables. In all comparisons, a *p*-value less than 0.05 was considered statistically significant.

## Results

Patients and control groups’ demographic characteristics are presented in Table 1. 25 patients (41.7%) had migraine with aura, and 35 (58.3%) had migraine without aura. The patients’ average daily water consumption was 1,550 cc. The average daily consumption of caffeinated beverages was found to be 800 cc. The patients’ average MIDAS score was 16.5 and their HIT-6 score was 57. Four patients (6.7%) worked night shifts that disrupted their sleep patterns. When evaluationing comorbidities in patients, hypothyroidism, hypertension, and type 2 diabetes were found to be the most common, respectively (11.7, 5, 3.3%). Only 1 of the 53 female patients was using oral contraceptives. When patients’ sleep patterns were assessed for frequent awakenings and difficulty falling asleep, 36 patients (60%) were found to have sleep disturbances. Patients who exercised regularly by walking at least 20 min 3 days a week were considered to be exercising regularly; these patients constituted 35% (21 patients) of the entire group. Patients were not asked about their family history of migraines.

**Table 1:** Demographic characteristics of the patient and control group.

Variable	Migraine patients (*n* = 60)	Control group (*n* = 30)	*p*
Gender (Female); *n* (%)	53 (88.3)	23 (76.7)	0.150*
Age (years)	32.5(25.0–41.0)	26.5 (22.0–34.3)	0.020**
BMI	26.1(23.1–30.0)	27.0(25.4–29.4)	0.433**
Cigarette	15(25)	13(43.3)	0.077*

Median (25–75), *n* (%); *Chi-Square Test; **Mann–Whitney *U* Test.

Netrin-1 and amylin levels were compared between episodic migraine patients and the control group, and in the presence of aura. In episodic migraine patients, the levels of molecules studied in serum samples taken during the headache attack period and the interictal period were found to be low in migraine patients regardless of whether they were in the attack period or the interictal period (Table 2).

**Table 2:** Comparison of netrin-1 and amylin levels between migraine patients and control group, and between the attack (ictal) period and the non-attack (interictal) period.

Variable	Group	Mean ± SD	Median (25th–75th percentile)	*p*
Netrin-1	Migraine (*n* = 60)	492.7 ± 638.0	173.6 (99.1–679)	**0.038**
	Control (*n* = 30)	744.8 ± 754.7	313.9 (168–1,566)	
Amylin	Migraine (*n* = 60)	324.9 ± 451.7	101.5 (42.9–453)	0.141
	Control (*n* = 30)	453.7 ± 490.2	171.9 (79.4–963)	
Netrin-1	Without aura (*n* = 35)	545.9 ± 720.6	172.3 (102–825)	0.922
	With aura (*n* = 25)	418.2 ± 504.9	190.9 (99.1–329)	
Amylin	Without aura (*n* = 35)	334.0 ± 463.7	102.5 (42.9–494)	0.863
	With aura (*n* = 25)	312.2 ± 443.5	97.4 (42.9–267)	
Netrin-1	Interictal (*n* = 35)	464.4 ± 558.3	164.3 (91.1–758)	0.393
	Ictal (*n* = 25)	532.2 ± 745.8	220 (124–514)	
Amylin	Interictal (*n* = 35)	308.8 ± 418.7	94.3 (36.2–494)	0.418
	Ictal period (*n* = 25)	347.5 ± 502.0	118 (73.7–340)	

^*^The Mann–Whitney *U* test was used. Netrin-1 levels are expressed in pg/mL and amylin levels in ng/mL. Mann–Whitney *U* test was used for comparisons.

**Figure 1:**
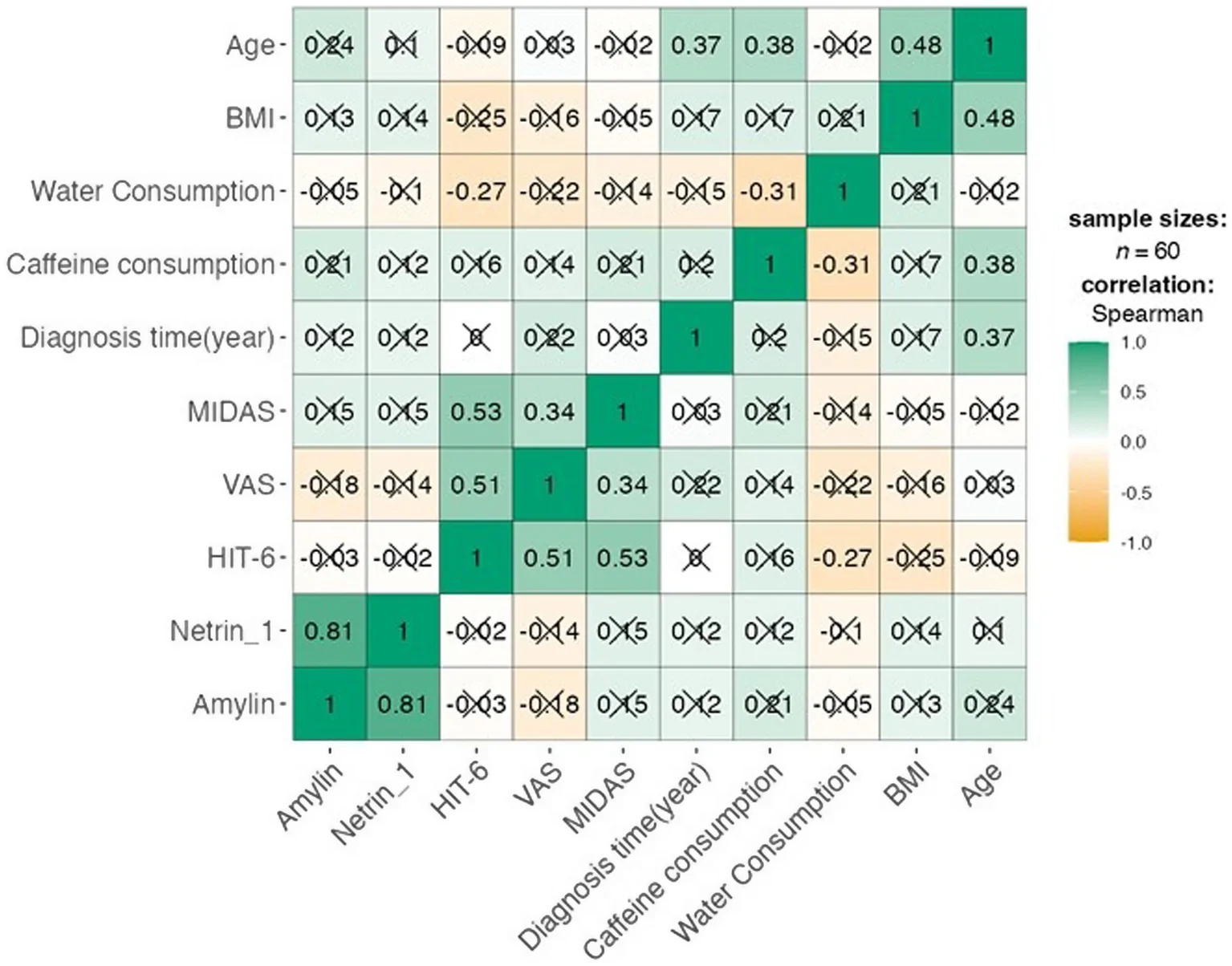
The relationship between netrin-1 and amylin levels and parameters such as age, BMI, clinical severity and daily living habits.

The relationship between netrin-1 and amylin levels and parameters such as age, BMI, clinical severity and daily living habits is shown in Figure 1. The results show that the scales used in the classification of headache severity and clinical classification are consistent with each other and with netrin-1 and amylin levels.

**Figure 2:**
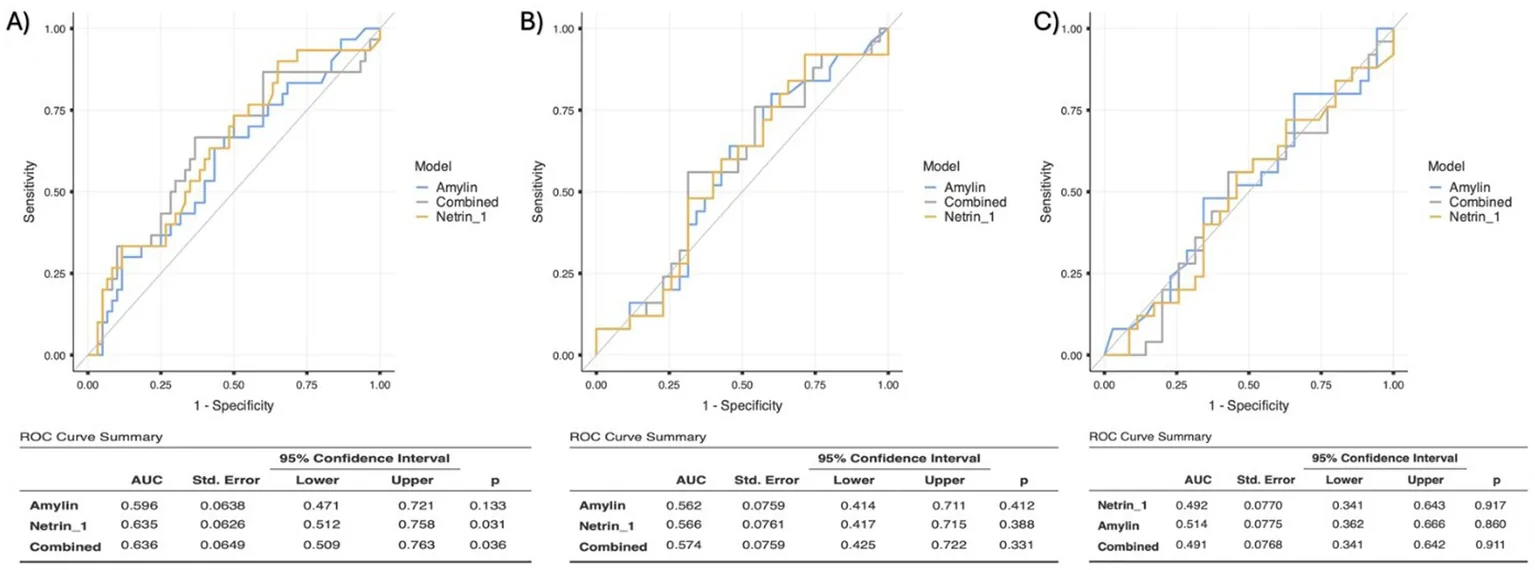
ROC curve analyses of serum netrin-1 and amylin levels for distinguishing migraine patients from healthy controls **(A)**, ictal versus interictal periods **(B)**, and migraine with aura versus migraine without aura **(C)**.

In the ROC curve analysis, serum netrin-1 levels demonstrated statistically significant diagnostic performance in distinguishing migraine patients from healthy controls. Although amylin levels alone did not show significant discriminatory performance, the combined model evaluating netrin-1 and amylin together also yielded significant results; however, the addition of amylin did not provide a substantial incremental contribution to the diagnostic performance of the model. In ROC analyses comparing ictal and interictal periods, no significant discriminatory performance was observed for netrin-1, amylin, or the combined model. Similarly, analyses comparing patients with migraine with aura and those without aura revealed no significant diagnostic performance for these biomarkers (Figure 2).

## Discussion

In our study, we examined the peptides netrin-1 and amylin, which are known to be associated with inflammation but had not previously been evaluated in episodic migraine. Serum netrin-1 levels were found to be markedly lower in patients with episodic migraine compared to the healthy control group. Although amylin levels were also lower in the migraine group, this difference did not reach statistical significance.

Serum netrin-1 levels in episodic migraine patients were found to be lower than those in the control group, with no difference observed between attack and interictal periods, and this difference was statistically significant. In addition, it was found that netrin-1 and amylin levels in patients with episodic migraine were lower during and between attacks compared to those in the control group. Furthermore, although no statistical significance was observed, when patients with aura were referred to those without aura, netrin-1 and amylin levels were similarly found to be lower in patients with aura than in those without aura.

Animal models’ experimental evidences investigating trigeminovascular activation strongly suggests that neuroinflammation plays a significant role in both episodic and chronic migraine, with and without aura. These findings are also supported by data from human studies. In particular, evidence is increasingly showing that plasma samples from migraine patients exhibit meaningful decreases in anti-inflammatory cytokines during both ictal and interictal periods referred to controls (13).

Inflammation stimulates Netrin-1 expression in endothelial cells of human blood–brain barrier, resulting in increased tight junction protein releasion and decreased blood–brain barrier permeability. A recent study examined the role of netrin-1 in inflammatory conditions; therapeutic and prophylactic administration of netrin-1 to mice with experimentally induced autoimmune encephalomyelitis stabilized the blood–brain barrier and reduced the severity of the disease. A previous study conducted on mice with induced autoimmune encephalitis demonstrated that intravenous infusion of recombinant netrin-1 supports the integrity of the blood–brain barrier and reduces neuroinflammation (14). Under inflammatory conditions, blood–brain barrier endothelial cells secreted markedly netrin-1 levels, thereby preventing BBB disruption and endothelial cell activation (14, 15). Furthermore, when studies on netrin-1 levels in neurological diseases are evaluated, it has been determined that netrin-1 levels, which have an anti-apoptotic effect in cerebral ischemia, decrease in ischemic stroke. It has been suggested that netrin-1 increases both vascular reperfusion in ischemic regions and neuronal regeneration may thereafter reduce endothelial cell apoptosis, increase vascular density, and reduce the infarct area (16). Additionally, in Alzheimer’s disease and mild cognitive impairment, serum netrin-1 protein levels were found to be low, and a correlation was observed between decreased dementia scores and decreased netrin-1 protein levels (17). Netrin-1 and its receptor are highly expressed, specifically in the dopaminergic neurons of the substantia nigra pars compacta. Netrin-1 decreases significantly in the brain with advancing age, and while this is likely caused by death of dopaminergic neurons, which are the primary source of netrin-1, a more pronounced decrease has been observed in the brains of Parkinson’s patients (18).

The netrin-1 molecule, previously known to play a role in inflammatory processes and shown to affect axon guidance and anti-inflammation (19). In migraine, data obtained by comparing netrin-1 levels during headache attacks, between attacks, and in the control group showed that netrin-1 levels were low in episodic migraine patients, regardless of whether they were during an attack or between attacks. This is important in demonstrating that inflammatory, cellular and molecular changes occur in the brain of migraine patients even in the absence of headache.

Considering other diseases in which netrin-1 levels have been studied, migraine has been found to be twice as common in inflammation-based diseases such as MS and rheumatoid arthritis compared to the normal population (20). Due to the inflammation-based pathogenesis of migraine, it is meaningful that netrin-1 levels are found to be low, similar to other diseases.

Aura formation is associated with hyperemia developing after oligemic cerebral cortex which is prominent in hypotheses, along with K^+^ channels, glutamate, NMDA receptors, serotonergic receptors, GABAergic receptors, and Ca^2+^ changes in astrocytes (21, 22). Due to the more severe inflammatory processes secondary to these ion changes, our study found that, similar to previous studies, clinical severity scores were markedly higher in patients with aura referred to those without aura. Therefore, clinical scores also showed greater severity, and netrin-1 levels were found to be lower in patients with migraine with aura referred to patients with migraine without aura.

The amylin molecule, which belongs to the CGRP family, has been evaluated in numerous animal and human studies due to its similarity to CGRP and its effect on common receptors. It has become a topic of current interest, particularly due to the recently proven efficacy of treatments targeting CGRP. A review of the literature reveals that the amylin molecule causes headache formation without being as effective as CGRP and, particularly due to its effect on AMY1 receptors, leads to headache clinical manifestations (23). Furthermore, the fact that amylin analogs cause severe headaches at a high rate, similar to patients receiving CGRP infusion, will guide the consideration of not only CGRP but also the amylin molecule in the treatment phase and the establishment of treatment targets for this molecule in conjunction with CGRP (8). The short half-life of amylin and crossing the blood–brain barrier ability, unlike CGRP, indicate that it is an important molecule that can be evaluated in pathophysiology. A review of the literature reveals that there is only one study in this field comparing chronic migraine patients with a control group (24). In this study, patients with episodic and chronic migraine were compared with a healthy control group, and it was found that serum amylin levels were elevated in patients with chronic migraine even outside of attack periods. The inclusion of patients with episodic migraine in our study sample may have contributed to the lower amylin levels observed in our study. Additionally, due to potential inaccuracies in determining the levels of common molecules at the receptor level and their concentrations in serum, amylin levels in our study may have been found to be lower than expected. Furthermore, amylin’s short half-life may also have influenced these results.

To determine whether changes in these inflammatory parameters play a role in the onset of the disease or whether the serum protein changes observed in the findings section are a result of the inflammatory process, it would be appropriate to assess the levels of these proteins in larger groups of patients with migraine. In this context, our study also aims to guide future research by evaluating these parameters for the first time in patients with episodic migraine.

### Limitations

Limitations of the study include feature a small cohort of patients and the fact that these values were not additionally evaluated in patients with new or previously known migraine. Furthermore, comparisons between the results of patients with netrin-1 and amylin levels during the attack period and those outside the attack period were not made on an individual basis; instead, serum values at the time of the study were taken into account. There are also differences between the control group and the patient group in terms of gender and age. Additionally, the single-center design of the study limited the evaluation of potential variations across diverse geographic or demographic populations.

## Conclusion

Understanding the molecular changes that process in the brain structural and functional in the pathophysiology of migraine and the cellular mechanisms that influence these changes plays a crucial role in identifying biomarkers reflecting inflammation-related changes in migraine patients after diagnosis, as well as in determining prognosis and treatment goals.

In our research, serum netrin-1 and amylin levels were evaluated in episodic migraine patients during attacks, between attacks, and in the control group. Netrin-1 levels were found to be lesser in episodic migraine patients referred to the control group, with no difference between attack and inter-attack periods. Furthermore, netrin-1 and amylin levels in episodic migraine patients were lower than in the control group both through and outside attacks. These findings may indicate that migraine patients experience changes in cerebral inflammatory and structural processes even outside of attacks, compared to healthy controls. However, it is important to evaluate these molecules comprehensively in conjunction with cellular-level receptors and factors that influence receptor binding. Further research on these parameters will yield more meaningful results.

## Data Availability

The original contributions presented in the study are included in the article/supplementary material, further inquiries can be directed to the corresponding author.
